# Bacilli on a Bald Head

**Published:** 1890-03

**Authors:** 


### BACILLI ON A BALD HEAD.

Dr. Saymonne claims to have isolated a bacillus, called by him "bacillus, crinivorax," which is the cause of alopecia. It is, he says, found only on the scalp of man- other hirsute parts of the body, and the fur of animals being free from it. The baccilli invade the hair-follicles and make the hair very brittle, so that they break off at the skin. Then the roots themselves are attacked. If the microbes can be destroyed early in the disease, the vitality of the hairs may be preserved; but after the follicles are invaded, and all their structures injured, the baldness is incurable. The following is Dr. Saymonne's remedy to prevent baldness: Ten parts of crude cod-liver oil, ten parts of the expressed juice of onions, and five parts of mucilage or the yolk of an egg, are thoroughly shaken together and the mixture applied to the scalp. and well rubbed in, once a week. This, he asserts, will certainly bring back the hair if the roots are not already destroyed: but the application of the remedy must be very distressing to the patient's friends and neighbors.

